# Combined Dorsal and Ulnarward Carpometacarpal Dislocation Associated with Open Fracture of the Base of First Metacarpal and Severe Degloving Injury

**DOI:** 10.1155/2017/7524563

**Published:** 2017-08-15

**Authors:** Ali J. Electricwala, Jaffer T. Electricwala

**Affiliations:** Electricwala Hospital and Clinics, Himalayan Heights, Pune, Maharashtra 411013, India

## Abstract

We report a rare case of dislocation of second to fourth carpometacarpal (CMC) joints of the right hand with combined dorsal and ulnarward displacement of the second to fourth digits and fracture of the shaft of the first metacarpal associated with degloving injury. These injuries were diagnosed early and treated successfully with closed reduction and internal fixation using Kirschner wires. The functional outcome was good at follow-up at 5 years. A high index of suspicion is required to successfully diagnose and treat this condition.

## 1. Introduction 

Carpometacarpal dislocation is an uncommon injury. A high-velocity injury in a vulnerable position of the hand is required. Such high-velocity injuries of the hand are seen commonly in boxers and motorcyclists. These injuries account for less than one percent of injuries of the hand and are frequently overlooked or missed [1–3]. Diagnosis of this rare form of injury requires careful examination and good radiography. Disability of the hand is severe in untreated individuals or in those where treatment is delayed [1]. We report such a case of combined dorsal and ulnarward carpometacarpal dislocation associated with open fracture of the base of first metacarpal and severe degloving injury in a 56-year-old adult male.

## 2. Case History

**Figure 1:**
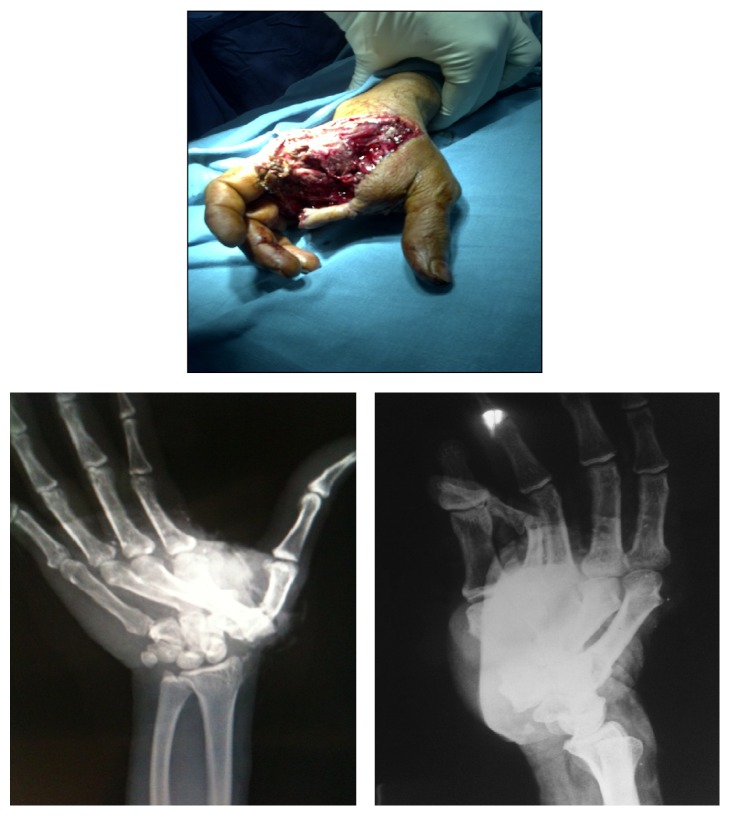
Clinical picture and X-ray at the time of injury.

A 56-year-old male met with a road traffic accident wherein an autorickshaw ran over his right hand. He was brought to our emergency department within half an hour of injury. On presentation, he was conscious, oriented, and hemodynamically stable. He had gross swelling over the dorsum of the right hand with a degloving wound approximately 10 × 5 cm in size, muscle deep on the palmar aspect extending from the first to fourth web space (Figure 1). He had no distal neurovascular compromise.

Radiograph of the right hand revealed dorsal and ulnarward CMC dislocation of second to fourth digits associated with a grade 2 compound fracture of the shaft of first metacarpal (Figure 1). The patient was operated on within two hours of injury.

**Figure 2:**
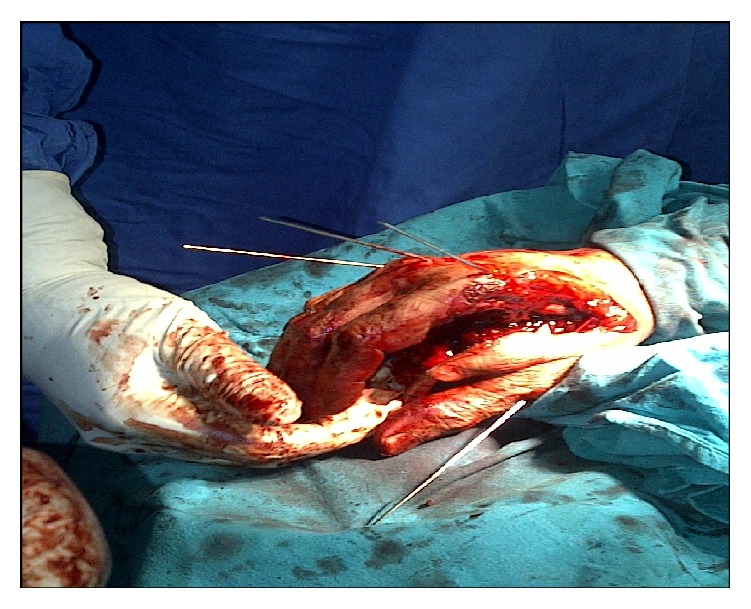
Intraoperative picture.

Closed reduction and internal fixation of all three CMC joints and fracture of first metacarpal were done using Kirschner (K) wires under image intensifier control (Figure 2). First “K” wire was passed from third metacarpal to capitate, second “K” wire from second metacarpal to trapezoid, third “K” wire from fourth metacarpal to hamate, and fourth “K” wire from first metacarpal to trapezium.

**Figure 3:**
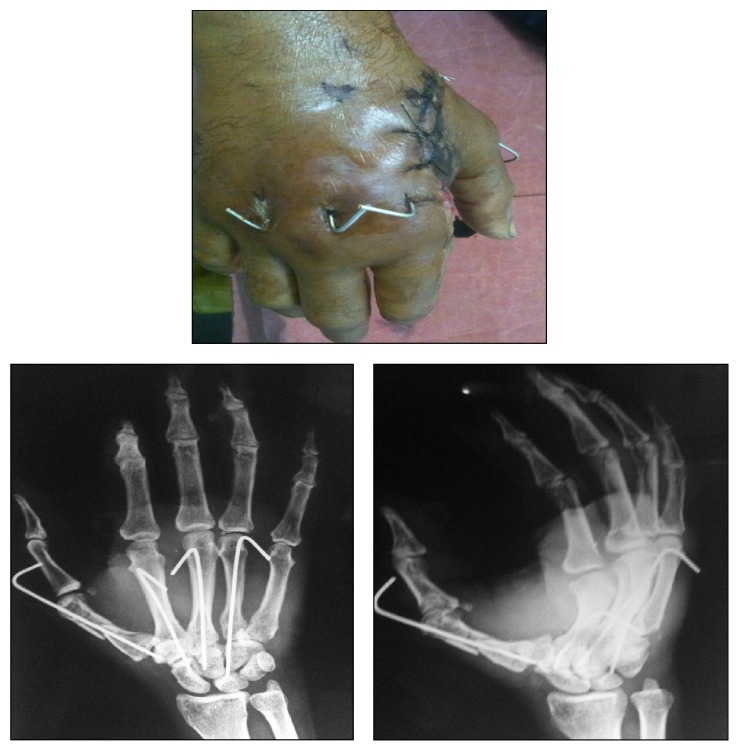
Clinical picture and X-ray at 2 weeks.

**Figure 4:**
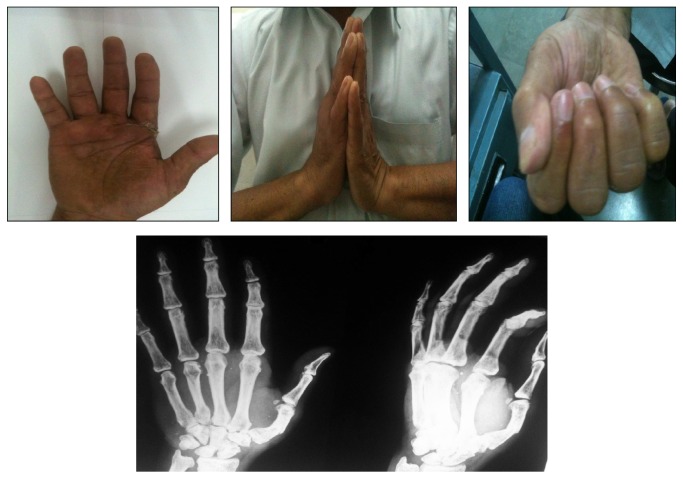
Clinical picture and X-ray at 3 months.

Thorough debridement of the wound was done. Devitalised tissue was excised. Primary closure was achieved over a drain. After stabilization with K wires, osseous anatomy and CMC joint congruency were restored (Figure 3). Operative time was one hour. Postoperatively, a below elbow cock-up splint was given till suture removal. Wound healed well after two weeks of regular aseptic dressings. K wires were removed after four weeks. Satisfactory reduction was maintained on reexamination at two months. At three months, the patient had a near full range of movement of the hand and wrist, showing good result (Figure 4). The functional outcome was good at latest follow-up at five years with no evidence of CMC joint arthritis.

## 3. Discussion

Carpometacarpal dislocation is a high-velocity injury. Stability in the finger's carpometacarpal joints is provided by a system of four ligaments. They are the dorsal carpometacarpal ligament, palmar carpometacarpal ligament, and the two sets of interosseous ligaments. In addition, the muscular-tendinous insertions function to further stabilize the carpometacarpal joints [4] (Table 1). The index metacarpal has a particularly stable configuration through its wedge-shaped articulation with the trapezoid [4–8]. These dislocations disrupt both the longitudinal and the transverse arches of the hand, resulting in an impaired grasp and loss of the normal axial length. Unreduced dislocations have definite functional disability with a decreased range of movement at the metacarpophalangeal joints and a poor grip [8].

It is important to diagnose and treat this injury very early to avoid considerable morbidity associated with this condition. Even though these injuries can be treated by different methods, better results are seen in closed reduction and internal fixation with K wires. Restoration of the third metacarpal-capitate-lunate axis is the key to good anatomical reduction. Open reduction and internal fixation with K wires are usually indicated in multiple dislocations, irreducible dislocations, old dislocations, and dislocations-associated fractures and in late presentations [1, 7]. For an acute single-injury pattern, closed reduction or closed reduction with K wire fixation is only indicated. It provides accurate reduction of the dislocation and early functional recovery [2, 3, 5, 6, 9].

## 4. Conclusion

Carpometacarpal dislocation resulting from traumatic event is rare. If missed, it may result in permanent hand disability. A high index of clinical suspicion and good radiographic imaging are essential to diagnose this condition. Early diagnosis and reduction of the dislocated carpometacarpal joint are the keys to restoring good hand function and preventing permanent disability.

## Figures and Tables

**Table 1:** Carpometacarpal articulations and muscular attachments.

Carpometacarpal articulation	Muscular attachment
Trapezoid and second metacarpal	Flexor carpi radialis
	Extensor carpi radialis longus
Capitate and third metacarpal	Extensor carpi radialis brevis
Hamate and fourth metacarpal	Nil
Hamate and fifth metacarpal	Extensor carpi ulnaris
	Flexor carpi ulnaris
